# Construction and Biological Evaluation of a Novel Integrin α_ν_β_3_-Specific Carrier for Targeted siRNA Delivery In Vitro

**DOI:** 10.3390/molecules22020231

**Published:** 2017-02-04

**Authors:** Xueqi Chen, Meng Liu, Rongfu Wang, Ping Yan, Chunli Zhang, Chao Ma, Lei Yin

**Affiliations:** Department of Nuclear Medicine, Peking University First Hospital, No. 8, Xishiku St., West District, Beijing 100034, China; xueqichen@bjmu.edu.cn (X.C.); pingyanzheng@126.com (P.Y.); zhangcl0326@163.com (C.Z.); machao00225@163.com (C.M.); jack-yin@163.com (L.Y.)

**Keywords:** RNA interference (RNAi), small interfering RNA (siRNA), arginine-glycine-aspartate (RGD), integrin α_v_β_3_

## Abstract

(1) Background: The great potential of RNA interference (RNAi)-based gene therapy is premised on the effective delivery of small interfering RNAs (siRNAs) to target tissues and cells. Hence, we aimed at developing and examining a novel integrin α_v_β_3_-specific delivery carrier for targeted transfection of siRNA to malignant tumor cells; (2) Methods: Arginine-glycine-aspartate motif (RGD) was adopted as a tissue target for specific recognition of integrin α_v_β_3_. To enable siRNA binding, a chimeric peptide was synthesized by adding nonamer arginine residues (9R) at the carboxy terminus of cyclic-RGD dimer, designated as c(RGD)_2_-9R. The efficiency of 9R peptide transferring siRNA was biologically evaluated in vitro by flow cytometry, confocal microscopy, and Western blot; (3) Results: An optimal 10:1 molar ratio of c(RGD)_2_-9R to siRNA was confirmed by the electrophoresis on agarose gels. Both the flow cytometry and confocal microscopy results testified that transfection of c(RGD)_2_-9R as an siRNA delivery carrier was obviously higher than the naked-siRNA group. The results of Western blot demonstrated that these 9R peptides were able to transduce siRNA to HepG2 cells in vitro, resulting in efficient gene silencing; and (4) Conclusion: The chimeric peptide of c(RGD)_2_-9R can be developed as an effective siRNA delivery carrier and shows potential as a new strategy for RNAi-based gene therapy.

## 1. Introduction

RNA interference (RNAi) has been developed as a significantly powerful and useful tool to block gene function through sequence-specific post-transcriptional gene silencing, particularly in cancer treatment. The efficacy of RNAi depends greatly on the effective delivery of small interfering RNA (siRNA) into target cells, where siRNA targets the homologous messenger RNA (mRNA) and results in the degradation of the latter by the RNA-induced silencing complex (RISC) [1]. 

Currently, a variety of viral and non-viral carriers are designed to transfer “naked” siRNA into target cells. Among them, cell penetrating peptides (CPPs) have received increasing attention because of their advantages in safety, non-immunogenicity, large-scale and standardized manufacturing, as well as improvements for transfer efficiency [2]. A short positively-charged peptide of 9-arginine (9R)—one component of CPPs—can deliver nucleic acids across the membrane bilayer through both energy-dependent endocytosis and energy-independent direct uptake, with high transfection efficiency and quite low cytotoxicity at a concentration range used for molecular delivery [2,3,4]. As CPPs are intended to function in a receptor-independent manner, a moiety of targeting ligands is always needed to attain the full potential of CPPs in specific studies. Although the mechanism of CPPs transferring siRNA is not fully understood, the importance of targeted ligands is being emphasized. According to the latest study [5], CPPs—including 9Rs—were of limited value when applied without the attachment to ligands in translocating siRNA. The reasons for this may involve membrane inversion and siRNA translocation at the site of receptor–ligand binding and late endosome-to-cytosol translocation mediated only by very high dose of ligand-9R. In some preclinical studies, 9R conjugated with targeting ligands have been reported to successfully transport siRNA to neuron cells, T-cells, and specific cancer cells [6,7,8,9].

To improve the siRNA transfection, we adopted an arginine-glycine-aspartate (RGD) ligand as a tissue target in the current study. RGD is reported to specifically recognize integrin α_ν_β_3_, which is well known to overexpress on the cell surface of activated tumor endothelial cells (TECs) and some types of cancer cells [10]. Although RGD peptide can bind to tumor cells, it does not combine, and therefore does not transport siRNA. The siRNA sequence we chose in this study was against the telomerase reverse transcriptase (hTERT), which is the catalytic subunit of telomerase and proved to be a universal and hopeful cancer therapy target of RNAi through specific inhibition of telomerase [11].

Above all, a chimeric peptide of cyclic RGD dimer targeting integrin α_ν_β_3_ was conjugated with 9R, designated as c(RGD)_2_-9R. Then, the efficiency of c(RGD)_2_-9R transferring siRNA was biologically evaluated in vitro.

## 2. Results

### 2.1. Structure of the Delivery Carrier and siRNA

**Figure 1:**
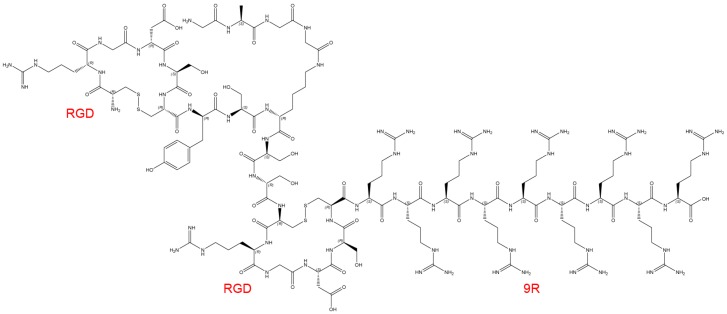
The chemical structure of c(RGD)_2_-9R, a chimeric peptide of cyclic RGD dimer targeting integrin α_ν_β_3_ conjugated with 9R. The chimeric peptide c(RGD)_2_-9R was synthesized by adding nonamer residues arginine (9R) at the carboxy terminus of cyclic-RGD dimer.

The peptide as a delivery carrier was synthesized by adding nonamer arginine residues (9R) at the carboxy terminus of cyclic-RGD dimer, designated as c(RGD)_2_-9R. The chemical structure of c(RGD)_2_-9R is shown in Figure 1. The design and optimization of the c(RGD)_2_ structure was described and evaluated previously [12]. The conjugated nonamer d-arginine residues allows the binding of siRNA through electrostatic interaction.

hTERT-targeted siRNA was against the sequence of hTERT mRNA, according to its significant inhibitory effect in previous studies [13,14]. The stable negative siRNA was also synthesized as control. For some in vitro experiments, siRNA with FAM (5-carboxy-fluorescein) labeled at the 3’ end of the sense strand was used. The sequences of siRNA against hTERT and negative siRNA are shown in Table 1.

### 2.2. Preparation of Delivery Carrier with siRNA

**Figure 2:**
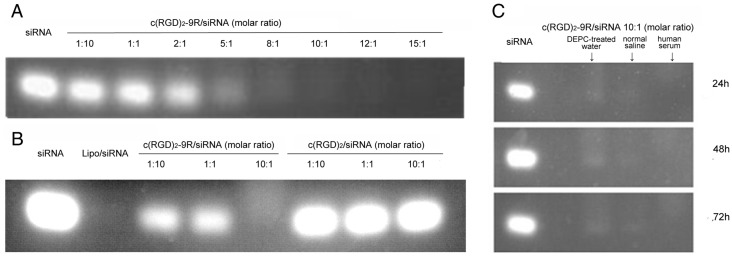
The combination of c(RGD)_2_-9R with siRNA. (**A**) siRNA was incubated with c(RGD)_2_-9R at the indicated molar ratios for 20 min. A minimal 10:1 molar ratio of peptide to siRNA showed to be optimal for maximal transduction; (**B**) siRNA was incubated with liposome, c(RGD)_2_-9R, or c(RGD)_2_ at the indicated molar ratios for 20 min. The position of the non-bound siRNA was indicated; (**C**) The combination of siRNA with c(RGD)_2_-9R was stable in diethylpyrocarbonate (DEPC)-treated water, normal saline at room temperature and 37 °C human serum at different time phases.

The c(RGD)_2_-9R peptide could bind siRNA in a dose-dependent manner, and its combination was verified in the electrophoretic gel mobility-shift assay. Different molar ratios of peptide to siRNA were incubated for 20 min. siRNA alone, incubated with c(RGD)_2_ (without 9R), or with Lipofectamine™ 2000 (liposome) served as controls. At a minimal molar ratio of 10:1 of peptide to siRNA, no free siRNA was detected (Figure 2A), as well as combination of liposome with siRNA as a positive control (Figure 2B). However, c(RGD)_2_ without 9R showed no ability to combine with siRNA at any molar ratios (Figure 2B). Consequently, it was determined that a 10:1 molar ratio of peptide to siRNA should be optimal for efficient siRNA transduction. 

We also tested the stability of c(RGD)_2_-9R/siRNA in diethylpyrocarbonate (DEPC)-treated water at room temperature, as well as in normal saline at room temperature and in fresh 37 °C human serum at 24 h, 48 h, and 72 h. The c(RGD)_2_-9R/siRNA as delivery complex remained stable in these three conditions after incubation (Figure 2C).

### 2.3. Flow Cytometry

**Figure 3:**
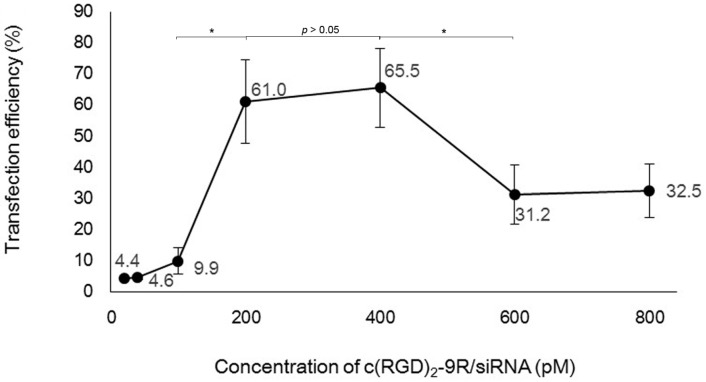
Transfection efficiency of siRNA delivery mediated by c(RGD)_2_-9R at different concentrations. * represented *p* < 0.05.

The HepG2 cells were cultured with the routine method. To determine the siRNA-transferring potency mediated by c(RGD)_2_-9R, the cells were transfected with a mixture of different concentrations of FAM-siRNA and c(RGD)_2_-9R (the molar ratio of peptide to siRNA was fixed at 10:1). After incubation with complexes for 6 h, the cells were collected and subjected to fluorescence-activated cell sorting (FACS). The results showed that transfection efficiencies were highest at the siRNA concentration of 200 pM and 400 pM (Figure 3). However, transfection efficiency did not improve significantly with 400 pM siRNA (65.5%) compared with 200 pM (61.0%). So, the concentration of 200 pM siRNA and its corresponding delivery carrier concentration were selected as optimal in the following tests.

**Figure 4:**
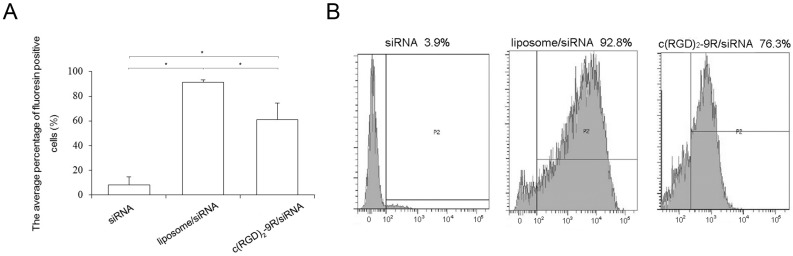
Flow cytometry analysis of cell transfection in vitro. HepG2 cells were transfected with FAM-siRNA alone, or FAM-siRNA mixed with liposome and c(RGD)_2_-9R at the concentration of 200 pM FAM-siRNA. The (**A**) average percentage and (**B**) representative plot of fluorescein-positive cells is indicated for each treatment. * represented *p* < 0.05.

Then c(RGD)_2_-9R/FAM-siRNA was added to HepG2 cells at this optimal concentration, along with FAM-siRNA mediated by liposome or naked FAM-siRNA as controls. After incubation for 6 h, the cells were also washed and analyzed by FACS. As shown in Figure 4, the average siRNA transfection efficiencies of c(RGD)_2_-9R and liposome were 61.0% ± 13.4% and 91.5% ± 1.84%, respectively, which were much higher than that of FAM-siRNA alone (8.08% ± 6.45%) (*p *< 0.05). 

### 2.4. Confocal Microscopy

**Figure 5:**
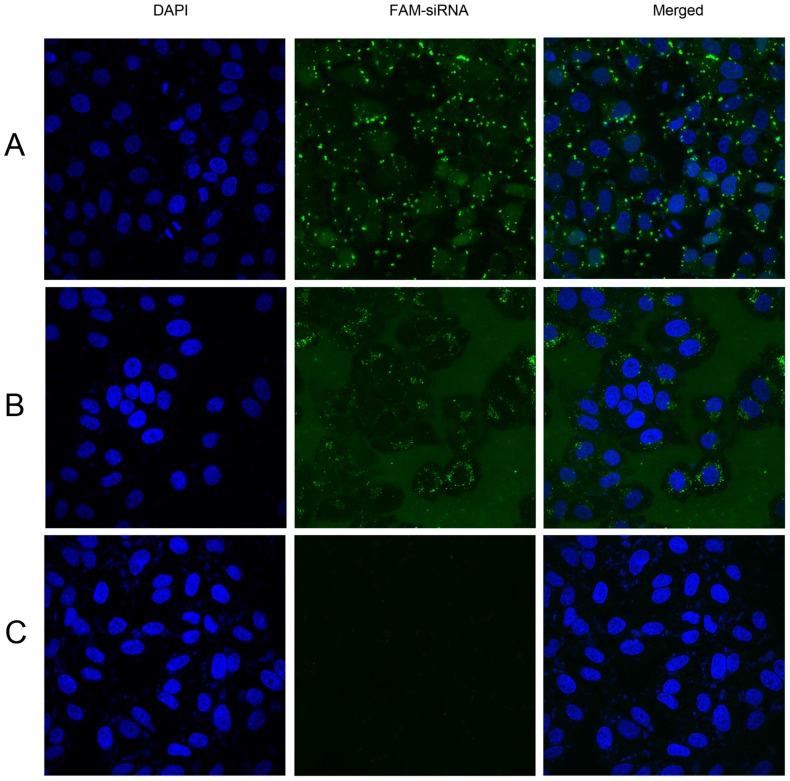
Confocal microscopy analysis of cell transfection in vitro. DAPI (4′,6-diamidino-2-phenylindole), FAM-siRNA, and merged images are displayed separately. HepG2 cells were treated with (**A**) FAM-siRNA/liposome, (**B**) c(RGD)_2_-9R/FAM-siRNA, and (**C**) FAM-siRNA alone, respectively. FAM-siRNA showed green fluorescence in the cytoplasm of HepG2 cells.

As we did for the flow cytometry, FAM-siRNA alone, liposome/FAM-siRNA, or c(RGD)_2_-9R/FAM-siRNA mixture were incubated and added to HepG2 cells. After transfection with complexes for 6 h, the cells were washed, fixed, and stained for confocal microscopy analysis to evaluate the transfection efficiency. As the results showed, green fluorescence signals (FAM-siRNA) were distinctly observed in the cytoplasm of HepG2 cells when treated with liposome/FAM-siRNA (Figure 5A), followed by c(RGD)_2_-9R/FAM-siRNA (Figure 5B). No obvious signal was observed when treated with FAM-siRNA alone (Figure 5C).

### 2.5. Western Blot

**Figure 6:**
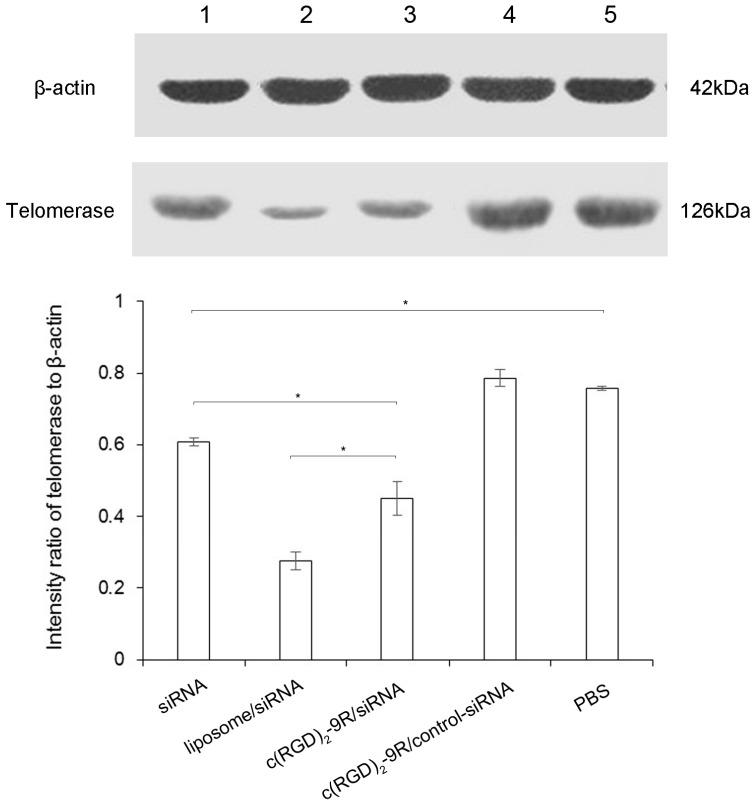
Western blot analysis of telomerase expression after transfection with different mixture: (1) naked siRNA only; (2) liposome/siRNA; (3) c(RGD)_2_-9R/siRNA; (4) c(RGD)_2_-9R/control-siRNA; (5) phosphate buffer saline (PBS) only. The band intensity ratios of telomerase to β-actin were also presented. * represents *p* < 0.05.

After transfected with mixture above for 72 h, the HepG2 cells were collected and followed with Western blot analysis. The telomerase protein expression was evaluated by Western blot to confirm the gene silencing ability of siRNA (Figure 6). The ratios of band intensity were calculated by telomerase to β-actin and presented as mean ± standard deviation. The most significant gene silencing was observed in siRNA transfected with liposome, followed by c(RGD)_2_-9R. No obvious gene silencing was observed in treatment with siRNA only, c(RGD)_2_-9R/control-siRNA, or phosphate buffer saline (PBS) only group.

## 3. Discussion

The major challenge in RNAi-based therapy is to develop an siRNA delivery carrier capable of protecting against nucleases, recognizing a desirable target, and penetrating the plasma membrane of target cells. To fulfil the maximal potential of RNAi, siRNA needs to be structurally modified and accompanied with a tissue-targeted delivery carrier.

Various types of chemically-modified siRNA could be classified into three groups; i.e., backbone, sugar, base and terminal modifications [15,16]. In this study, we used 2’-sugar modification by 2’-O-methyl (2’-OMe), which was able to improve nuclease resistance and reduce off-target effects. Because fully-modified siRNA with 2’-OMe could suppress silencing efficiency [17,18], we chose alternating 2’-OMe modification as an option. Besides, a deoxythymidine overhang at the 3’ end of each single strand was added to increase the stability and binding efficiency. These modifications gave siRNAs the ability to work efficiently in RNAi pathway.

Except for chemically-modified siRNA, there has been a concerted effort to bring forward more practical strategies for delivering siRNA in vivo, including viral and non-viral carriers. Non-viral carriers such as liposomes, nanoparticles, CPPs, and inorganic materials have become predominant because of their advantages in better manufacturability, immunogenicity, and safety [19,20].

Comprised of short amino acid sequences, CPPs can complex nucleic acids into nanoparticles and achieve intracellular access by crossing the membrane directly, or through the endocytic pathway. The major CPPs include penetratin, transportan, trans-activator peptide (TAT), poly-arginine, and protamine [2,4,21]. There are two approaches that use CPPs to deliver siRNA, one is based on covalent binding such as a disulphide linker, and the other is based on electrostatic complexation.

As a sort of CPP, 9R peptides attached to ligands are highly efficient in facilitating the cellular uptake of small nucleic acids. Kumar et al. [6] conjugated 9R with a short peptide derived from rabies virus glycoprotein (RVG) to successfully transport siRNA to the central nervous system of mice with Japanese encephalitis. In another study [7], they modified 9R with a CD7-specific single-chain antibody for T cell-specific siRNA delivery to effectively suppress HIV infection. In the present study, we conjugated 9R with a c(RGD)_2_ peptide to improve its siRNA translocation and targeting to the cell surface of TECs and some types of cancer cells. The peptides of c(RGD)_2_-9R recognize and interact with integrin by RGD sequence, and provide the ability of targeting to a variety of cancer cells where α_v_β_3_ integrin were overexpressed. Conjugating the carrier with a targeted or functional moiety is a useful and popular strategy for siRNA delivery, and has been validated in many preclinical studies [22,23,24].

It is noteworthy that the RGD sequence can not only work as the targeting moiety of the c(RGD)_2_-9R peptides, but may also assist the 9R delivery of siRNA into cells. One major route of cytosolic delivery starts with the recognition and binding of ligands to cell-surface receptor, which in our case means the RGD sequence binding to the integrin. Then, localized membrane inversion at the binding site occurs and the cellular entry proceeds, so that the translocation of siRNA across the membrane and diffusion within the cytoplasm are induced [5]. Therefore, both 9R and c(RGD)_2_ peptides play a vital role in the siRNA delivery.

For the optimization of the structure of RGD, we used dimeric and cyclized RGD peptides via double disulfide bonds to improve the properties of the peptides, such as binding affinity and selectivity. In consideration of the need for noninvasive visualization of siRNA delivery in vivo for further applications, we modified the peptides to allow radiolabeling. The tyrosine residue in the linker between two monomers could be used to radiolabel with ^125^I and ^131^I, while the tetrapeptide moiety of Gly-Gly-(D)Ala-Gly on the lysine residue could provide an N4 configuration for chelating with ^99m^Tc [12]. Our study showed that positively-charged c(RGD)_2_-9R could bind to siRNA through electrostatic interaction, when they were simply mixed for 20 min at room temperature. It has been reported that large amounts of cationic 9R peptide would increase the likelihood of non-specific interactions with anionic molecules and increase the difficulty of siRNA releasing from the endosome [4,21]. So, we determined the optimal molar ratios of c(RGD)_2_-9R to siRNA to be 10:1 based on the results of agarose gel electrophoresis. The complex of c(RGD)_2_-9R/siRNA remained stable even at 72 h under different conditions.

To determine the optimal concentration for c(RGD)_2_-9R transferring siRNA in HepG2 cells, we tested the transfection efficiency at different concentrations, and eventually selected the concentration of 200 pM for the following in vitro tests. When adding with a dose of siRNA higher than 600 pM, the efficiency decreased dramatically. It could be explained that the higher dose of siRNA delivered by a proportionally increased dose of c(RGD)_2_-9R might bring in excess delivery carrier or receptor saturation. 

The efficiency of c(RGD)_2_-9R as an siRNA delivery carrier was tested by flow cytometry and confocal microscopy. The flow cytometry results showed that the siRNA transfection mediated by c(RGD)_2_-9R was obviously higher than that of siRNA alone group, proving the delivery efficiency improvement using CPPs-ligands compared with non-carrier groups. In confocal microscopy, the fluorescence signals of FAM-siRNA could be observed in the transfection of c(RGD)_2_-9R, but not in the non-carrier group. Similar results were found in the gene silencing experiment at the protein level by Western blot. The transfection of siRNA mediated by c(RGD)_2_-9R led to an effective decrease of telomerase protein compared with non-carrier groups. The in vitro results above proved that the studied c(RGD)_2_-9R delivery carrier not only led to successful transfection, but also achieved effective downstream suppression. 

Although siRNA transfection mediated by commercial liposome performed better than c(RGD)_2_-9R in vitro, the potential cytotoxicity and non-target modification of the cationic lipids limited their application in vitro [25] and in vivo [26]; the toxicity issue of commercial liposomes has been one of the major drawbacks and has been commonly observed. In contrast, most current studies have found quite low or undetectable immune response and cytotoxicity within the CPPs concentration range used for molecular delivery [4,27]. What is more important, the targeting moiety inevitably fulfils the full potential of carriers in vivo, assisting in the binding of delivery carrier to the targeted cell surface. Therefore, these advantages of c(RGD)_2_-9R as a targeted delivery carrier may be stand-out features, and raise opportunities for future clinical application.

Based on what we discussed above, the construction of the c(RGD)_2_-9R as the delivery carrier of siRNA showed promise in vitro. For RNAi-based therapy to become clinically useful, delivery of siRNA to susceptible cells also needs to be examined in vivo in following studies. Additionally, with the increased potential of RNAi as a therapeutic strategy, new noninvasive methods for the visualization of siRNA delivery in vivo are urgently needed. Thus, the structure of c(RGD)_2_-9R peptide that has been designed to be radiolabeled could also be advantageous. 

## 4. Materials and Methods

### 4.1. Materials

The peptide of cyclic (RGD)_2_ conjugated with 9R (c(RGD)_2_-9R) was synthesized and purified at China Peptides Co., Ltd., Shanghai, China. hTERT-targeted siRNA, the stable negative siRNA, and FAM-siRNA for in vitro studies were synthesized by GenePharma Corp., Shanghai, China. All other chemicals were reagent grade and used without purification. 

### 4.2. Preparation of Delivery Carrier with siRNA

To verify the combination of c(RGD)_2_-9R with siRNA, 100 pM of siRNA was incubated with peptides at indicated molar ratios of peptide to siRNA (1:10, 1:1, 2:1, 5:1, 8:1, 10:1, 12:1, 15:1) for 20 min at room temperature, and then subjected to electrophoresis on (2%, *w*/*v*) agarose gels. Agarose gel containing ethidium bromide (EB) solution (0.5 μg/mL) was prepared in TAE (40 mM Tris-acetate, 1 mM EDTA) buffer (pH 8.0). siRNA alone or incubated with c(RGD)_2_ (without 9R) or Lipofectamine^TM^ 2000 (liposome) (Invitrogen, Carlsbad, CA, USA) served as control. Electrophoresis was conducted at 120 V for 25 min at room temperature, and the siRNA bands were visualized by UV illuminator (Champ Gel 1000, Sage Creation, Beijing, China). The stability of c(RGD)_2_-9R/siRNA in DEPC-treated water at room temperature, as well as in normal saline at room temperature and in fresh 37 °C human serum at 24 h, 48 h, and 72 h was confirmed by electrophoresis.

### 4.3. Cell Culture

The HepG2 cells were cultured in Dulbecco’s modified Eagle’s medium (DMEM) (Hyclone, Logan, UT, USA) supplemented with 10% fetal bovine serum (FBS) (GIBCO, Grand Island, NY, USA) and 100 mg/mL of penicillin-streptomycin (GIBCO). Cells were harvested by trypsin treatment (0.25% trypsin/0.02% ethylenediaminetetraacetic acid, 5 min, 37 °C).

### 4.4. Cell Transfection

Before transfection, the HepG2 cells were seeded in six-well plates with a density of 5 × 10^5^ cells per well, and incubated under standard condition for 24 h to permit adherence and growth (for liposome transfection group, the cells were cultured in Opti-MEM Reduced Serum Medium (Invitrogen) without antibiotics). On the second day, the cells were rinsed and cultured with fresh DMEM without FBS and penicillin-streptomycin. To determine the best concentration of transfection, the cells were transfected with a mixture of different concentrations of FAM-siRNA and c(RGD)_2_-9R (the molar ratios of siRNA to peptide was referred to the recommendation above), respectively. 

For further in vitro experiments, c(RGD)_2_-9R/FAM-siRNA was added to each well at a concentration indicated above. After incubation for 6 h, the cells were washed and incubated in fresh culture medium to maintain growth until additional analyses. As control, FAM-siRNA mediated by liposome or naked FAM-siRNA was also transfected into the cells. In the liposome/FAM-siRNA group, the transfection procedure was briefly described as follows according to the manufacturer’s instructions when the 200 pM siRNA was used for each well in six-well plates. Firstly, 200 pM siRNA and 10 μL liposome was diluted, respectively, in 500 μL medium and incubated for 5 min at room temperature. Then, we gently mixed the diluted siRNA with the diluted liposome and incubated for 20 min before adding to each well. The cells were also washed after 6 h and incubated in fresh culture medium like the other two groups.

### 4.5. Flow Cytometry

After incubation with complexes for 6 h, the cells were collected, washed, and incubated in tubes as a single-cell suspension, and then subjected to FACS (Beckton Dickinson, Franklin Lakes, NJ, USA) with a 495-nm laser and fitted with a high throughput sampler (HTS). The procedure started by gating out dead cells and scatter gating to remove small debris and large clumps of cells. Ten thousand cells (or “events”) were set up and recorded by the cytometer for each transfection group. Light emitted from FAM at around 520 nm wavelength was detected by the sensor for the FAM channel. The positive population was gated on and counted as the transfected cells. The ratio of transfected cells to all events was used as the transfection efficiency. All fluorescence data were analyzed using FACSDiva software (v6.0, BD Biosciences, San Diego, CA, USA).

### 4.6. Confocal Microscopy

After transfection with complexes for 6 h, the cells were carefully washed, then fixed with 4% paraformaldehyde for 30 min and permeabilized with 0.1% Triton-X-100 for 20 min. The coverslips were mounted after staining the nuclei with 4**′**,6-diamidino-2-phenylindole (DAPI) for 5 min and analyzed by confocal microscopy (Olympus Fluoview FV1000, Tokyo, Japan).

### 4.7. Western Blot

After transfected with mixture for 72 h, the HepG2 cells were collected and followed with Western blot analysis. The proteins were extracted with lysis buffer (20 mM Tris-HCl (pH 7.5), 150 mM NaCl, 1% Triton X-100, 1 mM EDTA, sodium pyrophosphate, Na_3_VO_4_, 1 mM Phenylmethanesulfonyl fluoride (PMSF), and protease inhibitor cocktail). The samples (30 μg of protein) were subjected to 10% sodium dodecyl sulfate polyacrylamide gel electrophoresis (SDS-PAGE), and transferred to polyvinylidene difluoride (PVDF) membranes (Millipore, Billerica, MA, USA). The membrane was incubated with anti-TERT rabbit monoclonal antibody [Y182] (1:1000, Sigma-Aldrich, St. Louis, MO, USA), β-actin (1:10,000, Sigma-Aldrich), then washed and incubated with horseradish peroxidase (HRP)-conjugated anti-rabbit antibody (GE Healthcare, Buckinghamshire, UK). The intensity of the bands was quantified using the Gel-Doc image system (Bio-Rad, Hercules, CA, USA).

### 4.8. Statistical Analyses

Statistical analyses were carried out using SPSS 17.0 (IBM Corporation, Armonk, NY, USA). Values of continuous variables were expressed as mean ± standard deviation. The means of continuous variables were compared with Student’s *t*-test. A *p*-value less than 0.05 was set to indicate statistical significance, and all tests were two-sided.

## 5. Conclusions

The chimeric peptide of c(RGD)_2_-9R can be developed as an effective siRNA delivery carrier, and its efficiency was validated in vitro. The peptides show potential as a new strategy for further carrier development, and can be examined in RNAi-based therapy in vivo.

## Figures and Tables

**Table 1:** Sequences of small interfering RNAs (siRNAs). FAM: 5-carboxy-fluorescein.

Name		Sequence
FAM-labeled siRNA	Sense	5’-FAM UUU CAU CAG CAA GUU UGG AdTdT-3’
(FAM-siRNA)	Antisense	5’-UCC AAA CUU GCU GAU GAA AdTdT-3’
Negative siRNA	Sense	5’-UUC UCC GAA CGU GUC ACG UdTdT-3’
	Antisense	5’-ACG UGA CAC GUU CGG AGA AdTdT-3’
